# Characterization of Prescription Patterns and Estimated Costs for Use of Oxygen Concentrators for Home Oxygen Therapy in the US

**DOI:** 10.1001/jamanetworkopen.2021.29967

**Published:** 2021-10-19

**Authors:** Peter A. Kahn, Christopher M. Worsham, Gretchen Berland

**Affiliations:** 1 Section of Pulmonary, Critical Care, and Sleep Medicine, Yale School of Medicine, New Haven, Connecticut; 2 Division of Pulmonary and Critical Care Medicine, Massachusetts General Hospital, Harvard Medical School, Boston, Massachusetts; 3 Section of General Internal Medicine, Yale School of Medicine, New Haven, Connecticut

## Abstract

This quality improvement study evaluates the electricity costs associated with the use of oxygen concentrators for home oxygen therapy among Medicare beneficiaries in the US.

## Introduction

Home oxygen is a critical component of therapy for patients with acute and chronic lung disease. Although there are several technologies available to deliver home oxygen, including compressed gas cylinders, liquid oxygen technologies, and oxygen concentrators, oxygen concentrators remain highly used due to their ease of use and no need for refills. Although the cost of oxygen concentrators and other durable medical goods is covered by Medicare, the cost of operating these devices is not. Despite the high prevalence of oxygen concentrators and expected increase in use due to the COVID-19 pandemic, minimal information is available about the financial burden of these devices and potential impact on device adherence.^1,2^ In this study, we sought to characterize prescription patterns for oxygen concentrators in the Medicare population as well as local energy costs for these devices.

## Methods

For this quality improvement study, we obtained the most recent Durable Medical Equipment data from 2018 for fee-for-service Medicare beneficiaries from the Centers for Medicare & Medicaid Services data warehouse. Data were filtered to retain only those Healthcare Common Procedure Coding System descriptions that included “oxygen concentrator,” which were combined for analysis.^3^ The number of total Medicare beneficiaries per state was obtained from the Kaiser Family Foundation.^4^ Annual electricity cost data were obtained from the US Energy Information Administration Electricity Data Browser for each state at residential delivery prices.^5^ Data regarding average watts used per hour were obtained by reviewing common user manuals from various oxygen concentrator models.^6^ For the purposes of generating estimates, concentrators were assumed to run 24 (continuous), 12, or 8 hours per day, 365 days per year. Annual electricity cost estimates were generated by multiplying the kilowatt-hour price of electricity by the wattage of the concentrator and the annual number of hours used. Analyses were conducted between March 4 and August 4, 2021, using R software. The Standards for Quality Improvement Reporting Excellence (SQUIRE) reporting guideline was followed in the preparation of this manuscript. As an analysis of aggregate and deidentified public use data, this study was not human subject research as defined by 45 CFR 46.102.

## Results

The cost of electricity varied substantially by state, resulting in wide variation across simulated oxygen concentrator costs in 2018 (Table). Hawaii, with the highest national cost of electricity, led the nation in this analysis with an annual projected maximal cost of $1991 for a high-oxygen-flow (>5 liters of oxygen flow per minute) 700-watt concentrator used continuously. A map of the US showing the estimated cost of running a regular-oxygen-flow 350-watt concentrator continuously for 1 year in each state is displayed in the Figure. States also varied significantly in the number of oxygen concentrators prescribed, ranging from 0.51% to 8.20% of the local Medicare population. Across states, median annual electricity costs ranged from $36 (low-flow 100-watt concentrators) to $751 (high-flow 700-watt concentrators).

**Table.** State-Level Estimated Annual Electricity Costs of Oxygen Concentrators

	Estimated annual operating cost, median (range) [IQR], US$^a^		
	24 h daily use	12 h daily use	8 h daily use
**Low-flow concentrators (1-3 LPM)**			
100-W models	107 (84-284) [97-122]	54 (42-142) [49-61]	36 (28-95) [32-41]
200-W models	214 (168-569) [195-244]	107 (84-284) [97-122]	71 (56-190) [65-81]
**Regular-flow concentrators (3-5 LPM)**			
150-W models	161 (126-427) [146-183]	80 (63-213) [73-92]	54 (42-142) [49-61]
350-W models	322 (252-853) [292-367]	161 (126-427) [146-183]	107 (84-284) [97-122]
**High-flow concentrators (>5 LPM)**			
400-W models	429 (336-1138) [389-489]	214 (168-569) [195-244]	143 (112-379) [130-163]
700-W models	751 (588-1991) [681-856]	375 (294-996) [340-428]	250 (196-664) [227-285]

Abbreviations: LPM, liters of oxygen flow per minute; W, watt.

^a^ State estimates of annual operating costs are based on average electricity costs for all 50 states and the District of Columbia, average electricity use of oxygen concentrator models, and average hours of daily use.

**Figure.**
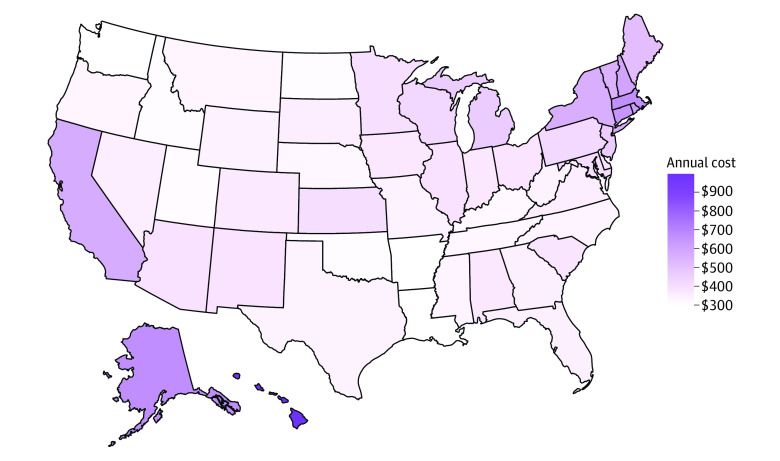
Estimated Electricity Cost of Continuous Use of a 350-Watt Oxygen Concentrator This map of the US shows the estimated cost of running a regular-flow (3-5 liters of oxygen per minute) 350-watt oxygen concentrator continuously (24 hours a day, 365 days a year) in each state, based on 2018 average electricity prices. Values range from $252 in Louisiana, where electricity prices are lowest, to $853 in Hawaii, where prices are highest.

## Discussion

This study’s findings suggest that, depending on the needs of the patient, local electricity prices, and the flow rate of oxygen prescribed, the cost of running electric oxygen concentrators can vary substantially and potentially result in financial hardship or suboptimal use of therapy because of cost concerns. Concentrator prescription patterns were highly variable by state. Factors such as altitude, prevalence of pulmonary disease, air quality, occupational exposures, and average age of beneficiaries likely contribute to prescribed oxygen flow rates and oxygen concentrator use patterns. Limitations of this study include lack of patient-level data, including concentrator use, health outcomes, utility service information, and nonelectricity costs to patients. Further study is needed to better characterize the relationship between durable medical equipment with persistently highly variable costs, such as oxygen concentrators; patient use patterns; and health outcomes.

State and federal policy makers should consider the implications of high-cost durable medical equipment and consider policy solutions to offset the cost of these devices. Particular consideration should be given to individuals with fixed or low income who receive these medical devices through Medicare and Medicaid programs. These patients are less likely to possess the resources to use the devices as intended without serious financial or health consequences, particularly if electricity is shut off due to nonpayment.
